# Is Skin-Touch Sham Needle Not Placebo? A Double-Blind Crossover Study on Pain Alleviation

**DOI:** 10.1155/2015/152086

**Published:** 2015-05-07

**Authors:** Miho Takayama, Hiroyoshi Yajima, Akiko Kawase, Ikuo Homma, Masahiko Izumizaki, Nobuari Takakura

**Affiliations:** ^1^Department of Acupuncture and Moxibustion, Faculty of Health Sciences, Tokyo Ariake University of Medical and Health Sciences, 2-9-1 Ariake, Koto-ku, Tokyo 135-0063, Japan; ^2^Department of Physiology, Showa University School of Medicine, 1-5-8 Hatanodai, Shinagawa-ku, Tokyo 142-8555, Japan; ^3^Japan School of Acupuncture, Moxibustion and Physiotherapy, 20-1 Sakuragaokacho, Shibuya-ku, Tokyo 150-0031, Japan; ^4^The Foundation for Oriental Medicine Research, 28-9 Sakuragaokacho, Shibuya-ku, Tokyo 150-0031, Japan

## Abstract

It remains an open question whether placebo/sham acupuncture, in which the needle tip presses the skin, can be used as a placebo device for research on pain. We compare the analgesic effect of the skin-touch placebo needle with that of the no-touch placebo needle, in which the needle tip does not touch the skin, in a double-blind crossover manner including no-treatment control in 23 healthy volunteers. The subjects received painful electrical stimulation in the forearm before and during needle retention to the LI 4 acupoint and after the removal of the needle and rated pain intensity using a visual analogue scale. We found no significant difference in analgesic effects among the skin-touch placebo needle, no-touch placebo needle, and no-treatment control at every point before, during, and after the treatments (*p* > 0.05). The results indicate that the skin-touch placebo needle can be used as a placebo device in clinical studies on pain.

## 1. Introduction

Acupuncture has been increasingly practiced in the Western world as an alternative medical therapy for pain management [1]. Although great numbers of clinical studies have been conducted with the aim of proving the efficacy of acupuncture, its efficacy has been controversial because safeguards against placebo effects were lacking in these studies [2, 3]. To control placebo effects in patients, single- or double-blind placebo needles with a blunt tip that presses the skin were invented [4–9]. The specific effect of acupuncture using penetrating needles over sham/placebo acupuncture has been failed to be demonstrated in the best controlled studies using such sham/placebo acupuncture needles [3]. Therefore, scientists have concluded that acupuncture with skin-penetrating needles does not have a specific effect over placebo acupuncture [3]. However, some researchers have questioned whether a placebo needle that touches the skin is true placebo [10, 11], whereas the blunt tip needles are considered to be ideal placebo acupuncture [12]. Thus, even if the efficacy of acupuncture using penetrating needles over skin-touch placebo/sham needles has not been revealed in good quality trials, it could not be concluded whether the penetrating needles have a specific effect over placebo or not [10]. It is ultimately necessary to develop a control device to skin-touch placebo needles to determine whether the skin-touch placebo needle is capable of being a safeguard against the placebo effect [13, 14].

We developed a skin-touch placebo needle that can be used to blind both acupuncturists and patients, which had been considered almost impossible to develop [8, 9]. Using these skin-touch placebo needles, we conducted a double-blind study on the analgesic effect of acupuncture to detect a specific effect of the penetrating needle [15]. In the previous study, we found that penetrating needle application failed to confer a specific analgesic advantage over skin-touch placebo needle application, whereas both the penetrating and skin-touch placebo needle trials resulted in a significant analgesic effect when compared with the no-treatment control condition [15]. However, we could not conclude whether the penetrating needle had a specific effect over the placebo effect because the skin-touch placebo needle is not physiologically inert and has analgesic effects [10, 11]. Therefore, we designed a no-touch placebo needle, that is, another version of the placebo needle, the tip of which does not touch the skin but which can still be matched to the validated double-blind skin-touch placebo and penetrating needles [13, 14] to solve this open question.

In the present study, we conducted a double-blind crossover study that compared the analgesic effects of skin-touch and no-touch placebo needle trials in healthy volunteers following the protocol of a previous pain study [15]. The aim of this study was to determine whether the skin touch with the blunt tip of a placebo needle had specific analgesic effects over no skin touch with the tip of a placebo needle under double-blind conditions.

## 2. Methods

### 2.1. Study Design

We conducted a crossover study in which the statistical significance was improved by eliminating most interpatient variances, as compared with the parallel-group designs that include more patients [15–17], to compare the analgesic effects of the skin touch with the blunt tip of a placebo needle, no skin touch with the tip of a placebo needle, and no-treatment control trials under double-blind conditions in healthy volunteers. The study was conducted at the Japan School of Acupuncture, Moxibustion, and Physiotherapy, Tokyo, Japan.

The study was approved by the Ethics Committee of Tokyo Ariake University of Medical and Health Sciences.

### 2.2. Participants

We recruited 23 eligible healthy volunteers (15 men, 8 women) from the Japan School of Acupuncture, Moxibustion, and Physiotherapy, who were familiar with acupuncture treatment. Their mean age was 33.4 (SD, 9.7) years. Exclusion criteria included subjects with any signs of neurological disorder, those ingesting painkillers or psychotropic drugs, and those with dermatological diseases. The purpose and format of the study were explained, and written informed consent was obtained from subjects before the study. One licensed acupuncturist participated as a practitioner.

### 2.3. Assignment

Each of the needles (23 sterilized skin-touch placebo needles and 23 no-touch placebo needles) was sealed in a small, sterilized opaque container. We prepared 23 opaque envelopes, one per subject, and each contained a skin-touch placebo needle, a no-touch placebo needle, and no needle. Nobody knew which container contained which needle in the envelope. Immediately before each trial, an assistant (blinded) took a container from the envelope to assign a skin-touch placebo needle, a no-touch placebo needle, or no-treatment control in a random order.

### 2.4. Intervention and Explanation

**Figure 1:**
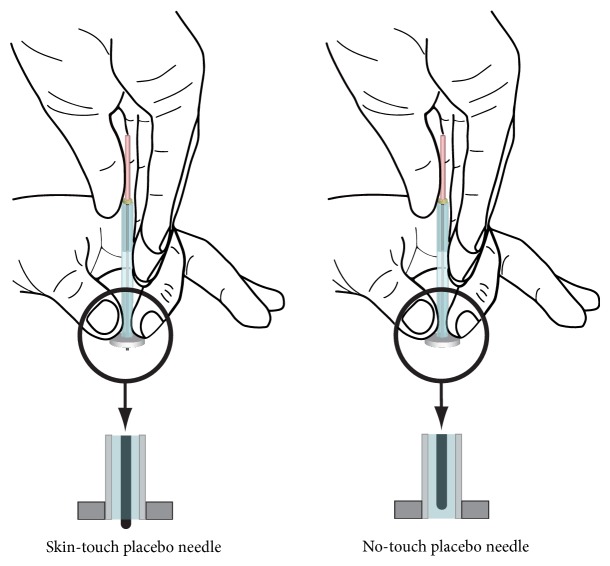
Illustrations of skin-touch placebo needle and no-touch placebo needle.

To detect a specific effect of the skin touch with the blunt tip of placebo needles, we used two types of needles for double blinding: (1) skin-touch placebo needles, the tip of which presses against the skin but cannot penetrate it, and (2) no-touch placebo needles, the tip of which does not touch the skin (Figure 1). These needles were designed to match the appearance and feel of the penetrating needles [13, 14]. The diameter of the needles was 0.16 mm. These details have been described elsewhere [8, 9, 13, 14].

Before the study began, participants were informed about the possible use of skin-touch placebo needles or no-touch placebo needles during the trials.

### 2.5. Pain-Eliciting Electrical Stimulation

Subjects reclined on a bed in the supine position with their right hands resting by the side of their bodies. A trained assistant delivered painful electrical stimulation to the middle of the posterior surface of the right forearm through surface electrodes using a constant-voltage isolation unit (SEN-3301, SS-104 J; Nihon Kohden Corp., Tokyo, Japan) [18–22]. The strength of the stimulation (square wave pulse: duration, 1 ms; interval, 1 s) that produced a clear sensation of pain (voltage, pain threshold ∗ 1.1-1.2) in each subject was determined before each trial of skin-touch placebo, no-touch placebo, and no-treatment control. The mean intensities for each of the three conditions did not differ significantly (skin-touch placebo needle trial, 56.5 ± 19.0 V; no-touch placebo needle trial, 55.9 ± 18.1 V; no-treatment control, 59.2 ± 18.0 V) (Friedman test, *p* = 0.73). Pain thresholds remained stable over time in individual subjects.

Twenty minutes before each needle application, the assistant delivered electrical stimulation (square wave pulse: duration, 1 ms; interval, 5 s) for 1 min to provide a baseline reading for pain. The assistant then delivered electrical stimulation for 1 min at the following times: 10 min before needle insertion, immediately after and 10 min after each needle application as well as 1 min before, immediately after, and 10, 20, and 30 min after the removal of the needle. Throughout the trial, subjects were blindfolded, except when they were asked to measure pain intensity from electrical stimulation or to measure pain from skin penetration and the* de qi* associated with needle application following the protocol of a previous study [15]. We asked subjects to measure pain intensity without application of the needles (no-treatment control) using the same methods and time intervals as those in the placebo needle trials.

### 2.6. Needle Application

For each needle trial, the acupuncturist applied the needle to the subject's right hand at the LI 4 point located in the middle of the 2nd metacarpal bone on the radial side, which is the most important analgesic point [18, 19, 23] on the large intestine meridian. We selected LI 4 based on a general principle that acupoints on the arms are usually used for treatment when the sites of pain are located in the upper arm [24]. Further, the most effective pain alleviation was obtained when acupoints governed by the same nerve innervating to the receptive field of pain were selected [25]. The acupuncturist inserted the needle using the alternating twirling technique (alternating between rotating the needle clockwise and counterclockwise) [8, 9, 13, 15].

The needle was left in place for 20 min [15, 19]. After 20 min, the needle body was returned to its initial position in an opaque tube. The entire needle assembly was removed from the skin and sealed in an opaque envelope. After each needle application, the acupuncturist was asked to record whether he thought the needle was “skin-touch placebo,” “no-touch placebo,” or “unidentifiable.”

Each trial was performed at about the same time on different days. To prevent any carryover analgesic effect [16, 17], the three trials were conducted more than 24 hours apart [18, 19].

### 2.7. Outcome Measures

The primary outcome measure was pain elicited by electrical stimulation to the posterior forearm. Immediately after each episode of painful stimulation, subjects were shown a visual analogue scale (VAS) ranging from 0 (no pain) to 200 [15, 26]. Subjects were asked to rate pain intensity 20 min before placebo needle application as baseline pain intensity and then rate each pain intensity, which was compared with baseline pain intensity (arbitrarily assigned a score of 100).

The secondary outcome measures were pain and the* de qi* associated with placebo needle application. Subjects rated pain and the* de qi* using a VAS ranging from 0 (no pain or* de qi*) to 100 (the most intense pain or* de qi*) [4, 9, 14].

### 2.8. Adverse Events

Despite the fact that we did not use penetrating needles, we asked subjects to report if they experienced any adverse event after placebo acupuncture treatment.

### 2.9. Statistical Analysis

We compared pain intensity scores for the three conditions (skin-touch placebo needle, no-touch placebo needle, and no-treatment control) using the Friedman test. We used the Kappa coefficient to measure the agreement between the practitioner's guesses regarding the treatments (excluding the “unidentified” responses) and the treatments.

## 3. Results

**Figure 2:**
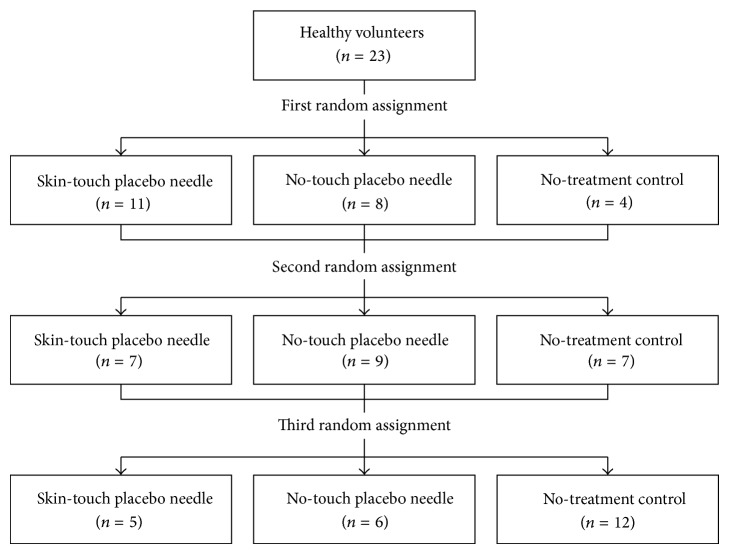
Flow of subjects through the study protocol.

The flow of subjects during the study is shown in Figure 2. All 23 subjects completed the study.

### 3.1. Pain Intensity

**Figure 3:**
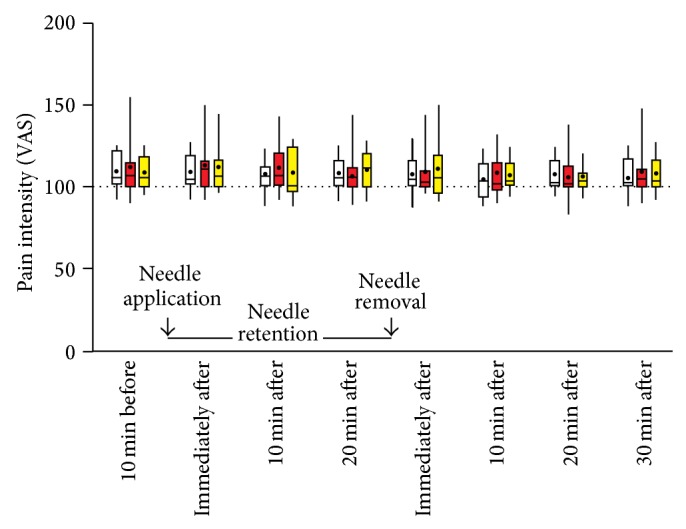
Changes in pain intensity rated by the 23 subjects before, during, and after application of the skin-touch placebo needles (red), no-touch placebo needles (yellow) and during the no-treatment control (white). The broken line (a score of 100) indicates baseline pain intensity measured at 20 min before needle application. The top, middle, and bottom lines of the boxes correspond to the 75th, 50th (median), and 25th percentiles, respectively. The whiskers extend from the 10th to the 90th percentile. The filled circles indicate the arithmetic mean.

We found no significant difference in the analgesic effects for pain in the right arm among the skin press with the tip of skin-touch placebo needles, no skin touch with the tip of no-touch placebo needles (to the ipsilateral LI 4), and no-treatment control measured at all the time points (Figure 3).

### 3.2. Pain and* De qi* with Placebo Needle Application

The median (mean ± standard deviation) intensity of needle pain for the five skin-touch placebo needles that elicited pain was 6.3 (7.7 ± 7.5); pain was not elicited by no-touch placebo needles.* De qi* intensity elicited was 2.8 for one skin-touch needle and 17.9 for one no-touch placebo needle.

### 3.3. Effect of Practitioner Blinding

The acupuncturist identified 15 needles correctly and 22 incorrectly and recorded 9 needles as “unidentifiable.” The Kappa coefficient between practitioner's guesses, excluding the “unidentified,” and the treatments was −0.201 (indicating “poor” strength of agreement [27]).

### 3.4. Adverse Events

No adverse events were observed during the experiment or were reported by subjects after the trials.

## 4. Discussion

In this double-blind study, we found that the analgesic effect of skin pressure from the skin-touch placebo needle was no greater than that from the no-touch placebo needle not to give a touch with the needle tip. Both of them had no analgesic effect compared with the no-treatment control, which showed the skin touch with the pedestal had no analgesic effect even though the skin touch has potential to induce a physiological response [11]. The skin-touch placebo needle was at least clinically inert for pain alleviation, even if physiologically active [11]. We believe placebo needles with a blunt tip can be used as placebo in studies on analgesic effects of acupuncture.

Traditionally, the definition of placebo is an inert substance or treatment lacking specific activity [28–31]. In recent randomized controlled acupuncture studies, placebo/sham needles with a blunt tip, which cannot penetrate the skin, were used in a placebo arm [32–34]. Further, placebo needles were used as placebo devices in many studies to investigate their placebo effect and its mechanisms [35–39]. Some researchers argued that sham/placebo acupuncture was not a true placebo because the pressing on the skin with the blunt tip of sham/placebo needles was not physiologically inert [10, 11]. There is no doubt that pressing the skin with the blunt tip of such sham/placebo needles activates the afferent fibers innervating the skin and finally the related brain regions. Placebo/sham devices that induce a certain amount of sensory stimulation have a possible therapeutic effect [10, 11]; could we say that touching or pressing the skin with something in daily life has a therapeutic effect because it provides a certain amount of sensory stimulation [10, 11]? The claim that the skin-touch placebo needle, which is not physiologically inert, is not placebo is untenable because it ignores the vast literature concerning the physiological effects of placebos and mind-body interactions [28–31, 40]. We consider that the salient point is to determine whether a placebo device has a specific effect, rather than determine whether the placebo device is physiologically inert [40]. In this sense, it can be said that the skin-touch placebo/sham acupuncture needle does not have a specific effect for pain alleviation in this study.

The distinctive feature of acupuncture is the penetration of the skin, which we believe has a distinctive meaning in acupuncture treatment. The skin-touch placebo needle does not have this feature of skin penetration. If the skin-touch placebo needle is another type of acupuncture having similar efficacy to that of acupuncture with a real penetrating needle [10, 11], there is no need for such an invasive tool as penetrating needles, which have potential risks [41]. To make a rational case for the use of such invasive acupuncture, the superiority of skin penetration to skin pressure with a blunt tip placebo needle must be verified. The skin-touch placebo needle has a scientifically important relevance in this sense, whether or not the skin-touch acupuncture needle is a real placebo.

We could not detect an analgesic effect of the skin-touch placebo needle over the no-treatment control in this study, an outcome different from the previous result that showed the skin-touch placebo needle had a significant analgesic effect over the no-treatment control [15]. Subjects informed about the possible use of the skin-touch placebo needle or the no-touch placebo needle in the present study might have less or no expectation regarding the received treatment than subjects informed of the possible use of real acupuncture in the previous study. The difference in analgesic effects between these studies suggests that the patient's expectation for receiving real penetrating acupuncture is a critically important factor in inducing an analgesic effect of genuine or placebo acupuncture even though verbal suggestion has weak placebo effects [42]. If the analgesic effect observed in the previous study comparing blunt tip needle use with no-treatment control was truly specific to skin stimulation with the blunt tip needle [15], the skin-touch placebo needles should have shown an analgesic effect over the no-touch placebo needles and the no-treatment control in this study. The present results indicate that the skin-touch placebo needle, although physiologically active, was an inert placebo for the analgesic experiment because the possible analgesic effect expected by the patient was excluded by informing subjects of the use of the skin-touch placebo needle or the no-touch placebo needle. Thus, a noninsertion acupuncture of Japanese style, that is, just skin touch or press, might not have a specific effect for pain alleviation. Further, these results suggest that the significant pain alleviation with the penetrating needle comparing with the no-treatment control reported in the previous study [15] might be produced by a nonspecific effect, which is consistent with the findings that a nonspecific effect of genuine acupuncture may play a significant role in the analgesic effect [42].

Our study has several limitations. First, the sample was relatively small because of resource constraints. Thus, we selected a crossover design, which has often shown greater statistical power than parallel-group designs with large samples [15, 43]. Second, there may have been a carryover analgesic effect of the treatment. To prevent a carryover effect, we designed the study so that there would be an interval of at least 24 hours between the two needle trials [18, 19]. This interval was rational when we considered the findings from a previous study [15], where alleviation of experimental pain with acupuncture at the LI 4 point was maintained for about 20 minutes after needle removal. We believe there was little carryover effect in this study because the significant pain alleviation was not detected in every arm, but further studies are necessary to determine the washout time of an acupuncture treatment to guarantee the quality of crossover acupuncture studies. Third, we did not ask subjects whether they received a skin-touch placebo or no-touch placebo needle so as not to induce bias in subjects in the second placebo needle application (by asking subjects' guesses at treatment in the first placebo needle application). Therefore, the successful blinding of subjects should be interpreted with caution, particularly for no-touch placebo needles. Even if the blinding was completely broken for the no-touch placebo needle, abolishing the patients' expectation for the no-touch placebo needle, the skin-touch placebo needle should have produced a specific effect for pain alleviation if the physiologically active skin-touch needle had a therapeutic effect. However, the analgesic effects of the skin-touch placebo and the no-touch placebo needles should be studied under conditions where subjects are not informed of the use of skin-touch and no-touch needles and informed exclusively of the use of penetrating needle or of the possible use of skin-touch, no-touch, and penetrating needles. Fourth, the theory of Chinese Medicine was not considered to choose acupoint. We chose the site to elicit pain and LI 4 to see analgesic effect according to the previous reports [18, 19] and the neurophysiological bases [24, 25]. We believe the results of this study should be the base to prove the validity of the theory of Chinese Medicine in future when it would be found that some acupoint chosen according to the theory of Chinese Medicine has the effect of pain alleviation in the arm. Finally, one thing we must note is that the skin is being touched with the pedestal of the no-touch placebo needles which has potential to induce a physiological response [11], although the analgesic effect of skin pressure with the pedestal of the no-touch placebo needle was not detected in this study.

## 5. Conclusion

A specific analgesic effect of the skin touch with the blunt tip of the placebo needle over the effects of the no skin touch with the tip of the placebo needle was not detected. The analgesic effect of skin pressure with the pedestal of the no-touch placebo needle was not detected. The results indicate that the skin-touch placebo needle can be used as a placebo device in clinical studies on pain.
